# Effects of Higher Dietary Protein and Fiber Intakes at Breakfast on Postprandial Glucose, Insulin, and 24-h Interstitial Glucose in Overweight Adults

**DOI:** 10.3390/nu9040352

**Published:** 2017-04-02

**Authors:** Akua F. Amankwaah, R. Drew Sayer, Amy J. Wright, Ningning Chen, Megan A. McCrory, Wayne W. Campbell

**Affiliations:** 1 Department of Nutrition Science, Purdue University, West Lafayette, IN 47907, USA; aamankwaah@calbaptist.edu(A.F.A.); amyjwright2@purdue.edu (A.J.W.); 2 Department of Public Health Sciences, College of Health Science, California Baptist University, Riverside, CA 92504, USA; 3 Anschutz Health and Wellness Center, University of Colorado—Denver | Anschutz Medical Campus, Aurora, CO 80045, USA; drew.sayer@ucdenver.edu; 4 Department of Statistics, Purdue University, West Lafayette, IN 47907, USA; chen929@purdue.edu; 5 Department of Health Sciences, College of Health & Rehabilitation Sciences: Sargent College, Boston University, Boston, MA 02215, USA; mamccr@bu.edu

**Keywords:** dietary protein, dietary fiber, breakfast, overweight, continuous glucose monitoring, meal tolerance test

## Abstract

Dietary protein and fiber independently influence insulin-mediated glucose control. However, potential additive effects are not well-known. Men and women (*n* = 20; age: 26 ± 5 years; body mass index: 26.1 ± 0.2 kg/m^2^; mean ± standard deviation) consumed normal protein and fiber (NPNF; NP = 12.5 g, NF = 2 g), normal protein and high fiber (NPHF; NP = 12.5 g, HF = 8 g), high protein and normal fiber (HPNF; HP = 25 g, NF = 2 g), or high protein and fiber (HPHF; HP = 25 g, HF = 8 g) breakfast treatments during four 2-week interventions in a randomized crossover fashion. On the last day of each intervention, meal tolerance tests were completed to assess postprandial (every 60 min for 240 min) serum glucose and insulin concentrations. Continuous glucose monitoring was used to measure 24-h interstitial glucose during five days of the second week of each intervention. Repeated-measures ANOVA was applied for data analyses. The HPHF treatment did not affect postprandial glucose and insulin responses or 24-h glucose total area under the curve (AUC). Higher fiber intake reduced 240-min insulin AUC. Doubling the amount of protein from 12.5 g to 25 g/meal and quadrupling fiber from 2 to 8 g/meal at breakfast was not an effective strategy for modulating insulin-mediated glucose responses in these young, overweight adults.

## 1. Introduction

Type 2 diabetes (T2D) is a chronic metabolic disease that is impacted by insulin resistance, glucose intolerance, and dyslipidemia [1,2,3]. Dietary factors that influence blood glucose control may modify these metabolic abnormalities [3,4,5,6]. Total dietary energy intake and macronutrient composition are well-known to modulate glycemia [4,7]. Restricting energy intake and the resultant reductions in body weight improves these modifiable risk factors for T2D [8,9,10]. Nevertheless, maintaining energy restriction and weight loss over the long-term is challenging [11]. Manipulating dietary macronutrient composition without restricting energy has also been shown to be an effective dietary strategy for prevention of T2D [12,13].

The quantity and quality of carbohydrate and protein in a mixed meal modulate postprandial glucose and insulin concentrations, with the total amount and the type of carbohydrate consumed being the major contributors to postprandial glucose concentration [14,15,16]. An increased amount of digestible/metabolizable carbohydrate consumed is associated with higher postprandial glycemia [17,18,19], which increases the risk of developing T2D [20,21]. On the other hand, a higher amount of complex/indigestible carbohydrates, such as soluble, viscous, and gel-forming fibers [22,23,24] may attenuate postprandial glucose concentrations [25,26,27,28,29]. Among different forms of soluble fibers, psyllium fiber is known to consistently attenuate fasting and postprandial glucose and insulin [30,31] and distinctly exert a laxation effect when consumed with meals [32]. Dietary protein appears to stimulate insulin secretion in people with normal glucose tolerance and T2D [33,34,35] and this insulinotropic effect of protein is related to secretagogue amino acids, such as arginine and leucine [33]. Eggs are a complete protein source with a relatively high leucine content [36]. Evidence supporting a glucose lowering effect of eggs is inconclusive [37,38,39]. Experimental evidence supports metabolic effects of protein and fiber in blunting postprandial and long-term glucose responses independently [7,12,13,35,40]. However, the extent to which the effects of protein and fiber intakes on postprandial glucose when co-consumed may be additive are uncertain, particularly in overweight individuals, who may be at risk of developing T2D.

While traditional meal tolerance tests adequately assess the impact of meals/diets on postprandial glucose and insulin responses, this method does not support the assessment of daylong glucose control. Continuous glucose monitoring (CGM) is an emerging technique that is used to determine the 24-h pattern of interstitial glucose concentrations in community-dwelling individuals [41,42]. The CGM technique may be preferred to inpatient meal tolerance tests to track the impact of dietary intake on daylong glucose responses, particularly when the impact of self-selected dietary intake on glycemia is of interest. By using this approach, the suggested extended favorable effects of protein and fiber intakes on daylong glucose control can be assessed.

We conducted a randomized controlled crossover trial to assess the independent and combined effects of normal versus higher egg-based protein and fiber intakes at breakfast on postprandial glycemic and insulinemic responses as well as 24-h glucose patterns with CGM. The study focused on breakfast because the typical intakes of protein and fiber at this meal are lower than at lunch and dinner [43]. In addition, some previous studies suggest that higher protein [13,44,45] or fiber [46,47] intake at breakfast, particularly protein, may have favorable metabolic benefits by enhancing insulin-mediated glucose disposal due to increased insulin secretion. We hypothesized that co-consumption of higher quantities of egg-based protein and fiber at breakfast would lower postprandial glucose and 24-h glucose responses.

## 2. Materials and Methods

### 2.1. Participants

**Figure 1:**
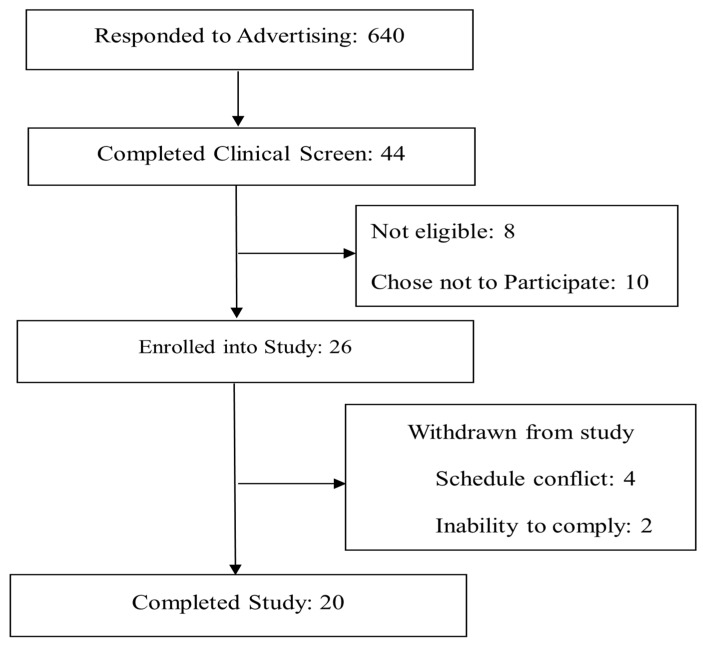
Consort diagram of the study.

Overweight male and female adults were recruited from the Greater Lafayette, Indiana, USA, area through advertisement to participate in the study. The inclusion criteria for selection of participants were: age 21–45 years; body mass index (BMI) 25–29.9 kg/m^2^; females with regular menstrual cycles; non-smoking; not taking medications or dietary/herbal supplements known to affect energy regulation or appetite; not pregnant or lactating within the past 1 year or planning a pregnancy; weight stable (±3 kg) for the past 3 months; not following a vigorous exercise regimen or weight loss program within the past 6 months; no acute illness; not diabetic (fasting blood glucose ≤126 mg/dL (7 mmol/L); not severely claustrophobic; willing to eat study foods; and not skipping breakfast >2 day/week. Eligible individuals were approved by the study physician to participate based on routine blood chemistry assessments. All participants gave written informed consent and the study had approval from the Purdue University Biomedical Institutional Review Board. Figure 1 shows participants’ flow through the study. Baseline participant characteristics were not statistically different (*p* > 0.05) between groups of participants who did versus did not complete the study. Two male participants were withdrawn from the study after randomization by the investigators due to an inability to fully comply with study procedures. These participants did not find the CGM comfortable to wear (one after three days of wearing the device and the other at the end of first intervention period). Four participants had scheduling conflicts. One of these individuals was not able to show up for the first test day appointment. The other three withdrew from the study after completing one or two testing periods. Data for these three participants were not included in the analysis. We obtained and included data from the 20 study completers in the postprandial glucose and insulin analysis. However, one of these 20 participants was unable to wear the CGM because it interfered with his farming occupation. This trial was registered on ClinicalTrials.gov (NCT02169245).

### 2.2. Experimental Design

Participants completed this randomized crossover trial in approximately 15 weeks. Participants consumed four breakfast meals that varied in protein and fiber amounts daily for 2 weeks in random order. The breakfast types were normal protein and fiber (NPNF), normal protein and high fiber (NPHF), high protein and normal fiber (HPNF), and high protein and fiber (HPHF). During each 2-week intervention period, participants consumed the designated breakfast type every morning, but foods and beverages outside of breakfast were otherwise uncontrolled and self-chosen. Testing days were scheduled to occur on the last day of each 2-week intervention period. Twenty-four-hour interstitial glucose patterns were assessed by CGM during the second week of each 2-week intervention period. On the last day of each intervention period, a meal tolerance test was performed. Participants arrived at our clinical research facility after a 10-h overnight fast. Upon arrival, a fasting blood draw was obtained. Participants next consumed the same breakfast type (NPNF, NPHF, HPNF, and HPHF) that they had eaten for the preceding 2-week dietary intervention period. Participants were asked to consume the entire breakfast in no more than 15 min. Postprandial blood draws were obtained hourly for 4 h after the completion of breakfast. Fasting and postprandial serum samples were analyzed for glucose and insulin concentrations. Each 2-week period of dietary intervention was separated by ≥15 days (mean: 17 days, range: 15–36 days) during which time the participants were asked to consume their habitual diets. Study investigators (with the exception of the research dietitian, AJW) and participants were blinded to the protein and fiber content of the breakfast treatments during the study data collection period.

### 2.3. Breakfast Characteristics

All breakfast recipes (Tables S1–S16 were designed by a research dietitian (AJW) using ProNutra (Release 3.2, Viocare Technologies, Inc., Princeton, NJ, USA) and were prepared by metabolic research kitchen staff in conjunction with the NIH-supported bionutrition center at Purdue University. Breakfasts were provided to the participants to consume either on-site or at a self-chosen location. Four varieties (egg and potato casserole, quiche, breakfast sandwich, and breakfast burrito) of each breakfast type were provided within each 2-week intervention period to provide variety. Within each breakfast variety, pilot testing was completed to ensure comparable palatability and textural characteristics across NPNF, NPHF, HPNF, and HPHF breakfasts. While all breakfast varieties were designed to achieve the similar energy, macronutrient, and fiber amounts, the breakfast burrito was consumed on all testing days to provide additional consistency (Table 1).

All four breakfast types (NPNF, NPHF, HPNF, and HPHF) provided ~400 kcals of metabolizable energy and contained ~50 g of available carbohydrate. High and normal protein were defined as 25 g and 12.5 g of protein, respectively. The increases in breakfast protein content for HPNF and HPHF were achieved by the addition of whole eggs and/or egg products such as egg white powder; and fat content was reduced to maintain similar total energy content among breakfast types. The high and normal fiber intakes were defined as 8 g, and 2 g, respectively. The additional fiber in NPHF and HPHF was achieved by adding powdered psyllium husk to the meals, which was shown to reduce postprandial glucose responses [30,31] and can easily be incorporated into meals for participant and investigator blinding. The energy, protein, and fiber content of the NPNF breakfasts were designed to resemble reported breakfast characteristics for adult males and females as described in the 2013–2014 What We Eat in America data tables [43]. The protein contents of HPNF and HPHF were doubled to 25 g (25% of energy at breakfast), which is consistent with published definitions of HP diets [48,49].

### 2.4. Dietary Intake

Self-selected food and beverage intakes during each intervention period were determined using the multiple-pass approach on three unscheduled days (one weekend day and two non-consecutive weekdays) and analyzed by the research dietitian using Nutrition Data System for Research (NDSR) software, version 2013 (Nutrition Coordinating Center, Univ. MN, Minneapolis, MN, USA). Two-dimensional food portion visuals (Nutrition Coordinating Center, Univ. MN, Minneapolis, MN, USA) were used to assist subjects in estimating portion sizes.

### 2.5. Body Composition

Standing height without shoes was measured during baseline with a wall-mounted stadiometer. Whole body mass, fat mass, and lean mass were also determined at baseline using air displacement plethysmography (BOD POD, COSMED USA, Concord, CA, USA). Body mass index was calculated as body mass divided by height squared (kg/m^2^).

### 2.6. 24-h Glucose (CGM)

A Medtronic iPro2 Professional CGM device (Northridge, CA, USA) was used to obtain 24-h continuous interstitial glucose concentration data from study participants during the last 7 days of each dietary intervention period. The glucose oxidase-based sensor inserted into the abdominal area at least 5 cm away from the umbilicus obtain an interstitial glucose measurement every 10 s and a recorder stored a smoothed and filtered average of these values every 5 min. Data obtained on the first and last day while participants wore the CGM device were excluded to obtain 24-h glucose data within periods where CGM sensor functional life was ideal. We considered 24-h glucose data valid when three or more self-monitoring glucose reading (finger sticks) were documented for calibration of CGM sensor glucose data [50]. CGM data were used to calculate 24-h interstitial glucose peak, mean, coefficient of variation (CV) and total area under the curve (AUC). The group mean and peak glucose were calculated from the individual participants’ means of 2–5 days of useable CGM data. Similarly, group CV was calculated as mean of the ratio of the individual participants’ standard deviation to mean. Also, in a subset of participants, the CGM data were used to document the postprandial glucose responses for 75 min after participants consumed the same trial-specific breakfast test treatment (breakfast burrito) as consumed during the corresponding meal tolerance test and also consumed their next meal or beverage by 75 min after consuming the test-specific breakfast treatment. Only participants who provided these data (obtained on different days) were used to graphically show the postprandial glucose responses in more detail (interstitial fluid samples every 5 min) to complement blood sampling (serum samples every hour) during the meal tolerance tests.

### 2.7. Biochemical Analyses and Calculation

Each blood sample was collected into vials containing serum separator and silica clot activator that were inverted several times and maintained at room temperature for 45 min to allow clotting and then centrifuged at 4 °C for 10 min at 4400 rpm. The resulting serum aliquots were stored at −80 °C until thawed for measurements of glucose by enzymatic colorimetry using an oxidase method on a COBAS Integra 400 analyzer (Roche Diagnostic Systems USA, Indianapolis, Indiana) and insulin by an electrochemiluminescence immunoassay method on the Elecsys 2010 analyzer (Roche Diagnostic USA, Indianapolis, Indiana). The trapezoidal method [51] was used to calculate total AUC for glucose and insulin at 0–120 min, 120–240 min, and 0–240 min time periods. The homeostatic model assessment (HOMA) insulin resistance (HOMA-IR), HOMA β-cell function (HOMA-%β) and whole-body (composite) insulin sensitivity index (ISI) were calculated as previously described [52,53].

### 2.8. Statistical Analysis 

Independent *t*-test was used to assess baseline metabolic health indices between the individuals who completed vs. those who did not complete the study. Doubly repeated-measures ANOVA (PROC MIXED) was used to assess the effects of protein intake (normal vs. high), fiber intake (normal vs. high), and time (fasting-state (0) vs. 60 vs. 120 vs. 180 vs. 240 min), protein × time, fiber × time, and protein × fiber × time interactions on serum fasting and postprandial glucose and insulin. The 2 repeated factors in our model were time and intervention period. Repeated-measures ANOVA (PROC MIXED) was used to assess the effects of breakfast meal treatments on 0–120, 120–240, and 0–240 min AUCs; 24-h interstitial glucose peak, mean, and CV, and predictive indices of glucose control measures HOMA-IR, HOMA-%β, and ISI. Data are presented as unadjusted mean ± standard deviation (SD) for participant characteristics and mean ± standard error of the mean (SEM) for all other results. We adjusted for fasting-dependent variable values (only when the outcome variable was expressed as AUC), sex, breakfast treatment order (chronological testing order: Period 1 vs. Period 2 vs. Period 3 vs. Period 4), and intervention carryover effect, as previously described [54]. Post hoc analyses were performed using Tukey–Kramer adjustment for multiple comparisons. Statistical significance was set using α = 0.05, two-tailed. Statistical Analysis Systems software, version 9.3 (SAS Institute Inc., Cary, NC, USA) was used to perform all statistical analyses.

## 3. Results

### 3.1. Participant Characteristics

Participants were apparently healthy young overweight adults (seven females, 13 males). Baseline fasting glucose, lipid and lipoprotein concentrations were within the clinically normal reference ranges (Table 2).

### 3.2. Breakfast Treatments Effect on Outcome Measures

#### 3.2.1. The Effect of Breakfast Treatment on Fasting Glucose and Insulin Variables

Fasting serum glucose, insulin, and predictive indices of glucose control including HOMA-IR, ISI, and HOMA-β (%) measured on the last day of each two-week breakfast treatment were not different among treatments (Table 3).

#### 3.2.2. Time Course of Postprandial Glucose and Insulin Responses (AUC)

**Figure 2:**
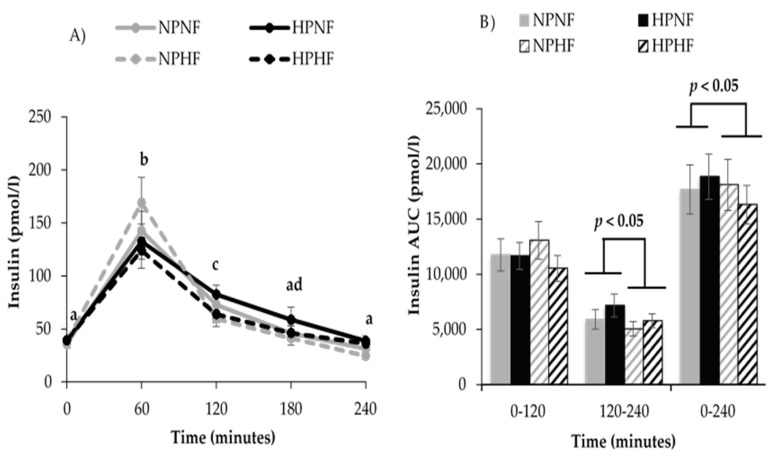
Postprandial time course (**A**) and total area under the curve (AUC) (**B**) for insulin response to breakfast treatments. Postprandial time points with different letters are statistically different (main effect of time, *p* < 0.05). *n* = 20, Estimates are unadjusted mean ± SEM. The main effects of protein (high vs. low) and fiber (high vs. low) and their interaction on postprandial insulin were assessed using a repeated-measures PROC mixed effects model, adjusting with fasting glucose (AUC only), for sex, dietary treatment order, and carryover effect. An effect was observed for fiber at 120–240 min (*p* = 0.002) and 0–240 min (*p* = 0.030) but not protein at 120–240 min (*p* = 0.113) or 0–240 min (*p* = 0.569) or their interaction (*p* = 0.944). NPNF: Normal Protein + Normal Fiber; HPNF: High Protein + Normal Fiber; NPHF: Normal Protein + High Fiber; HPHF: High Protein + High Fiber.

**Figure 3:**
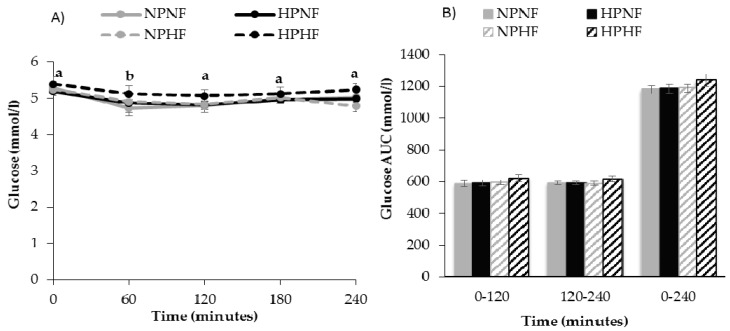
Postprandial time course (**A**) and total area under the curve (AUC) (**B**) for glucose response to breakfast treatments. *n* = 20, Estimates are unadjusted mean ± SEM. Values without a common letter are different, *p* < 0.05. The main effects of protein (high vs. low) and fiber (high vs. low) and their interaction on glucose were assessed using a repeated-measures PROC mixed effects model, adjusting for fasting glucose (AUC only), sex, dietary treatment order, and carryover effects. No effect or interaction effect was observed. NPNF: Normal Protein + Normal Fiber; HPNF: High Protein + Normal Fiber; NPHF: Normal Protein + High Fiber; HPHF: High Protein + High Fiber.

**Figure 4:**
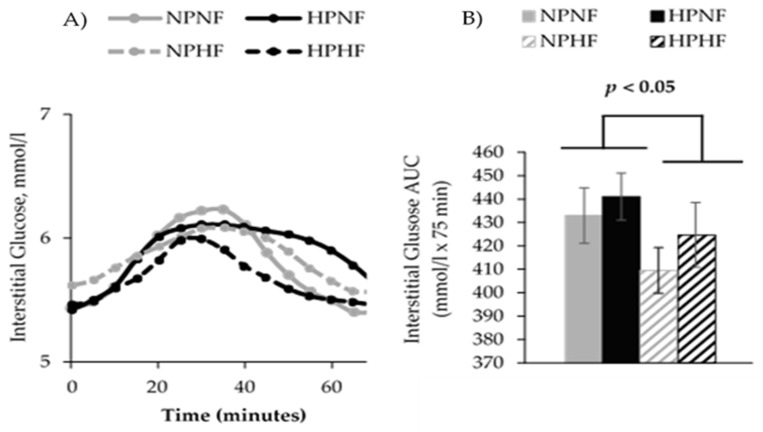
Postprandial time course (**A**) and total area under the curve (AUC) (**B**) for continuous glucose monitoring (CGM)-measured interstitial glucose after treatment-specific breakfast test meals (breakfast burrito) were consumed. Estimates are unadjusted mean ± SEM. The main effects of protein (high vs. low) and fiber (high vs. low) and their interaction on postprandial interstitial glucose were assessed using a repeated-measures PROC mixed effects model, adjusting with fasting glucose (AUC only), for sex, dietary treatment order, and carryover effect. An effect was observed for fiber (*p* = 0.017) but not protein (*p* = 0.631) or their interaction (*p* = 0.795). NPNF: Normal Protein + Normal Fiber; HPNF: High Protein + Normal Fiber; NPHF: Normal Protein + High Fiber; HPHF: High Protein + High Fiber. NPNF: *n* = 11, HPNF: *n* = 15, NPHF: *n* = 14, HPHF: *n* = 14.

Results from meal tolerance testing showed that over time, postprandial glucose (*p* = 0.005) and insulin (*p* < 0.0001) responses occurred. Compared to fasting (time 0), insulin was higher at 60 and 120 min and glucose was lower at 60 min. The protein and/or fiber contents of the breakfast treatments did not affect 240-min glucose AUC. Protein intake did not affect 240-min insulin AUC, while higher fiber intake lowered 240-min insulin AUC (*p* = 0.030). Further analysis of the insulin AUCs indicated that higher fiber intake reduced insulin AUC at 120–240 min (*p* = 0.002), but not at 0–120 min (*p* = 0.245) (Figure 2 and Figure 3). The apparent lack of a postprandial rise in glucose prompted an assessment of CGM-based interstitial glucose data from days that participants consumed the same treatment-specific breakfast burritos. Indeed, interstitial glucose increased after consumption of each breakfast meal treatment, peaking after about 30 min, and decreasing to baseline concentrations by 60–75 min (Figure 4). An effect was observed for fiber (*p* = 0.017) but not protein (*p* = 0.631) or their interaction (*p* = 0.795).

### 3.3. 24-h Interstitial Glucose Variables

Among the four breakfast treatments, we did not observe differential responses in the interstitial glucose 24-h peak (*p* = 0.768), mean (*p* = 0.255), and CV (*p* = 0.534) and AUC (*p* = 0.179) (Table 4). Comparable 24-h interstitial glucose profiles were observed for all breakfast treatments (Supplementary Figure S1).

### 3.4. Daily Energy and Macronutrients Intake

Among the four breakfast treatments, we did not observe differential responses in either the non-breakfast energy intake (self-chosen energy intake) (*p* = 0.421) or daily energy intakes (breakfast energy + self-chosen energy intakes after breakfast meals) (*p* = 0.394) (Supplementary Table S17 and S18), respectively. While we did not observe differences with the non-breakfast macronutrients intakes (Supplementary Table S17), we did observe differences in the daily total dietary fiber (*p* = 0.021), soluble fiber (*p* < 0.0001), and polyunsaturated fatty acids (*p* = 0.015) intakes but not the other macronutrients assessed (Supplementary Table S18).

## 4. Discussion

We conducted this study with the primary aim to assess the combined effect of higher intakes of protein and fiber consumed daily at breakfast over a 2-week period on postprandial glucose and insulin responses. We hypothesized that increasing protein and fiber amounts at breakfast would blunt postprandial serum glucose responses compared to increasing either protein or fiber alone. Contrary to our hypotheses, we observed that increasing protein and fiber amounts at breakfast did not influence postprandial glucose responses in overweight young adults. However, we observed an effect of fiber on insulin response (AUC). Increasing fiber lowered both the last two hours (120–240 min) and the composite 240-min insulin AUCs. Also, we did not observe protein and/or fiber breakfast meal effects on 24-h interstitial glucose or indices of glucose control. 

Results from the meal tolerance tests suggested the lack of a postprandial glucose response that may be explained by the timing of blood sampling in the current study. Specifically, it is likely that postprandial glucose concentrations peaked and began returning to baseline levels before the first postprandial sample 60 min after breakfast. This is supported by interstitial glucose data obtained via CGM that showed peak glucose concentrations approximately 30 min after breakfast and declining to baseline concentration by 60–75 min. Generally, the expected postprandial glucose peak occurs one hour postprandial in overweight and obese adults [46]. This supported our choice of hourly blood sampling for the meal tolerance testing because our study participants were overweight adults. Other studies that included varying amounts of fiber [55] and protein [44,56] to meals also reported shorter postprandial glucose peaks occurring within 30–45 min but declining to either baseline or below baseline glucose concentration by 60 min. However, a modestly elevated postprandial insulin at 60 min was concurrently reported by those studies with meal tolerance testing. Our results are consistent with such studies. We observed that glucose concentration 60 min after breakfast was slightly, but statistically, lower than the fasting serum glucose concentration. Our results, together with others, suggest that future studies must always use frequent blood sampling (15-min interval within the first hour of feeding), particularly when both fiber and protein amounts are varied in test meals, to possibly avoid missing important data points within the first hour after feeding.

Studies assessing whether the consumption of higher amounts of protein and fiber simultaneously would better attenuate postprandial glucose response compared to when either protein or fiber is increased and consumed separately are lacking. To our knowledge, only one study assessed the effects of a moderate protein (from egg source) and high fiber (whole grains) breakfast treatment (protein = 20 g, fiber = 7 g, carbohydrates = 45 g) compared with a refined rice cereal breakfast (protein = 10 g, fiber = 1 g, carbohydrate = 55 g) [57]. Bonnema et al. [57] reported an attenuation of 3.5-h AUC glucose with the moderate protein and high fiber breakfast. Results of insulin AUC were not reported. Of note, the difference in carbohydrate content of those breakfasts may explain the glucose results as opposed to the combined effect of protein and fiber. In contrast, one previous study [58] systematically assessed the individual and combined effects of low vs. high glycemic index (GI) and protein breakfast treatments on postprandial insulin and glucose responses. Makris et al. [58] found that combining higher amounts of protein and low GI simultaneously did not have an effect on postprandial insulin and glucose responses. However, intake of higher amounts of the low GI breakfast meal attenuated postprandial insulin and glucose responses. We also did not observe an additive effect with intake of higher amounts of protein and fiber on postprandial insulin and glucose. Collectively, the expected favorable additive effect with increasing the amount of protein and either low GI carbohydrates or fiber from whole grains sources and fiber per se on postprandial insulin and glucose responses is inconclusive.

Our results partially supported the favorable metabolic effects established for soluble viscous fibers. The 6-g difference between the normal fiber (2 g) and high fiber (8 g) breakfast treatments was achieved by adding psyllium husk powder, which is known as a soluble fiber. We observed that increasing soluble fiber intake attenuated the postprandial insulin response. Other studies noted that increasing soluble fiber when a meal had greater amounts of high glycemic index foods/metabolizable carbohydrate content attenuated postprandial glucose [55,59] and insulin [55] responses. The lowering of insulin with increasing dietary fiber intake that was observed in our study is consistent with previous findings [27,55,60]. Reducing postprandial insulin with higher fiber intakes has beneficial metabolic effects in healthy overweight individuals who are at risk of developing hyperinsulinemia, an early abnormality preceding the development of type 2 diabetes. Insulin resistance with concurrent compensatory hyperinsulinemia is a major feature that underlies the development of metabolic morbidities, particularly, type 2 diabetes [61]. This compensatory elevation of insulin secretion is usually needed to achieve a normal postprandial glucose response in overweight and obese individuals [62] after a meal. Intervention strategies that alleviate the need for elevated insulin secretion for glucose clearance are beneficial. While dietary interventions that vary dietary macronutrient composition without inducing weight lost do not consistently improve insulin resistance, manipulating the dietary macronutrient content has been documented to impact the insulin resistance-induced hyperinsulinemia [46]. Therefore, increasing dietary fiber supported improvement in insulin sensitivity (lower insulin concentration to normalize blood glucose) as previously established [24,47].

In addition to assessing postprandial glucose and insulin responses by meal tolerance testing, our study design allowed us to evaluate potential “second meal effects” with CGM. Jenkins et al. [63] defined second meal effects as an effect of a first meal on the postprandial glucose response after eating the second meal. Consuming higher amounts of fiber and protein were previously shown to elicit “second meal effects” by reducing postprandial glucose responses following a second meal [64,65,66,67]. Other studies also reported an attenuation of daylong glucose (lunch and dinner meals) with intake of low GI foods [68]. Assessing such a prolonged effect of consuming a breakfast with controlled protein and fiber amounts on subsequent and uncontrolled meals was an important and novel aim of the current study. However, 24-h interstitial glucose variables, including AUC, were not influenced by the 4 breakfast treatments used in the current study, which suggests a lack of a “second meal effect”. Our results may be due to the observed large variability between participants’ self-selected intake after the breakfast meal (Tables S17 and S18). Our findings could also suggest that the inclusion of fiber in multiple meals across the day may be needed with the intake of a typical Western diet to improve daily glycemic control. 

One limitation of the study was that the lack of provision of a standard meal the night before the acute measurement to metabolically stabilize our study participants may have confounded our study outcomes. Previous studies suggest a residual effect from dinner meals may impact the next morning glucose profile [68] particularly with high fiber intake. Another limitation was the fact that we could not match sugar, MUFA, PUFA, and total fat, across normal vs. the high breakfast treatment meals although these nutrients may influence our results. However, the magnitude of the differences in the amounts of the afore-mentioned nutrients between the normal vs. high protein and fiber breakfast treatments where not numerically substantial. Lastly, our study findings are only generalizable to overweight young adults. A strength of this study is that both researchers and study participants were blinded to the study dietary treatment. This potentially eliminates any biases with our dietary manipulation that may influence our study outcomes. Another strength is that the 2-week dietary acclimation period before our postprandial responses were assessed allowed us to determine the prolonged effects of consumption of protein and fiber breakfasts beyond a single meal.

## 5. Conclusions

In conclusion, consumption of a breakfast meal with both higher protein and fiber content did not differentially influence postprandial glucose response and 24-h glucose peak and AUC compared to the other three breakfast treatments. However, increasing fiber had an attenuation effect on the postprandial insulin response. Therefore, doubling the amount of protein from 12.5 g to 25 g/meal and quadrupling fiber from 2 to 8 g/meal simultaneously at breakfast may not be an effective therapeutic strategy to improve insulin and glucose responses acutely; however, higher fiber intake at breakfast may be effective for lowering the postprandial insulin response in healthy overweight, young adults.

## Figures and Tables

**Table 1:** Energy and macronutrient distribution of provided breakfast treatments.

Dietary Variables	Breakfast Treatments			
	NPNF	HPNF	NPHF	HPHF
* Energy (kcal)	396	397	387	386
Available Carbohydrate (g)	51	50	51	48
Sugar (g)	18	22	11	14
Total Fiber (g)	2	2	8	8
Soluble Fiber (g)	0	1	6	7
Insoluble Fiber (g)	2	1	2	1
Total Protein (g)	12.5	25	12.5	25
Total Fat (g)	16	10	14	10
Saturated Fat (g)	4	3	4	3
Monounsaturated Fat (g)	6	3	6	3
Polyunsaturated Fat (g)	3	1	2	1
Trans Fat (g)	0	0	0	0
Cholesterol (mg)	114	325	114	325
Sodium (mg)	767	723	765	720

* Metabolizable energy. Data are based on information from ProNutra, Release 3.2, Viocare Technologies, Inc., Princeton, NJ, USA. NPNF: Normal Protein + Normal Fiber; HPNF: High Protein + Normal Fiber; NPHF: Normal Protein + High Fiber; HPHF: High Protein + High Fiber.

**Table 2:** Baseline participant characteristics.

Variable	Mean ± SD
Age (years)	26 ± 5
Height (cm)	175 ± 10
Body Mass (kg)	83.4 ± 10.2
BMI (kg/m^2^)	27.0 ± 1.3
% Body Fat	26.4 ± 9.5
Serum glucose (mmol/L)	5.2 ± 0.3
Total cholesterol (mmol/L)	4.3 ± 0.5
Low-density lipoprotein cholesterol (mmol/L)	2.6 ± 0.5
High-density lipoprotein cholesterol (mmol/L)	1.2 ± 0.2
Triglycerides (mmol/L)	1.1 ± 0.4

Values are means ± standard deviation (SD) for *n* = 20 participants. BMI: body mass index.

**Table 3:** Fasting serum glucose, insulin, and indices of glucose control after two weeks of breakfast meals consumption.

Fasting Variables	Breakfast Treatments				
	NPNF	HPNF	NPHF	HPHF	*p*
Glucose (mmol/L)	5.3 ± 0.2	5.3 ± 0.2	5.2 ±0.1	5.2 ± 0.2	0.924
Insulin (pmol/L)	36 ± 6	36 ± 6	42 ± 6	42 ± 6	0.695
HOMA-IR	1.39 ± 0.1	1.51 ± 0.2	1.55 ± 0.2	1.54 ± 0.2	0.713
ISI	33 ± 5.2	32 ± 4.7	30 ± 5.6	31 ± 4.3	0.803
HOMA-β (%)	79 ± 10.2	79 ± 9.5	115 ± 36.9	88 ± 18.2	0.740

*n* = 20. Estimates are mean ± SEM. Homeostatic model assessment (HOMA) insulin resistance (HOMA-IR), HOMA β-cell function (HOMA-%β); whole-body (composite) insulin sensitivity index (ISI). The main effects of protein (high vs. low) and fiber (high vs. low) and their interaction on these fasting variables were assessed using a repeated-measures PROC mixed effects model, adjusting for sex, dietary treatment order, and carryover effect. NPNF: Normal Protein + Normal Fiber; HPNF: High Protein + Normal Fiber; NPHF: Normal Protein + High Fiber; HPHF: High Protein + High Fiber.

**Table 4:** Breakfast treatment effects on 24-h interstitial glucose variables.

Glucose Variables	Breakfast Treatments				
	NPNF	HPNF	NPHF	HPHF	*p*
Peak (mmol/L)	7.4 ± 0.2	7.1 ± 0.2	7.2 ± 0.1	7.2 ± 0.2	0.768
Mean (mmol/L)	5.5 ± 0.1	5.5 ± 0.1	5.5 ± 0.1	5.2 ± 0.1	0.255
Variability (CV)	0.99 ± 0.54	0.33 ± 0.23	0.30 ± 0.24	0.60 ± 0.38	0.534
AUC (mmol/L × 1440 min)	7982 ± 109	7977 ± 123	7755 ± 109	7860 ± 104	0.179

*n* = 19. Estimates are mean ± SEM. CV, coefficient of variation. AUC: total area under the curve. The main effects of protein (high vs. low) and fiber (high vs. low) and their interaction on 24-h interstitial glucose variables were assessed using a repeated-measures PROC mixed effects model, adjusting for sex, dietary treatment order, and carryover effect. No effect was observed for fiber and protein or their interaction. NPNF: Normal Protein + Normal Fiber; HPNF: High Protein + Normal Fiber; NPHF: Normal Protein + High Fiber; HPHF: High Protein + High Fiber.

## Supplementary Materials

The following are available online at www.mdpi.com/2072-6643/9/4/352/s1, Figure S1: 24-hour time course interstitial glucose from CGM to breakfast treatments and self-selected intakes after provided breakfast. *n* = 19, Estimates are unadjusted means. NPNF: Normal Protein + Normal Fiber; HPNF: High Protein + Normal Fiber; NPHF: Normal Protein + High Fiber; HPHF: High Protein + High Fiber, Table S1: NPNF breakfast burrito recipe, Table S2: HPNF breakfast burrito recipe, Table S3: NPHF breakfast burrito recipe, Table S4: HPHF breakfast burrito recipe, Table S5: NPNF breakfast sandwich recipe, Table S6: NPHP breakfast sandwich recipe, Table S7: NPHF breakfast sandwich recipe, Table S8: HPHF breakfast sandwich recipe, Table S9: NPNF breakfast casserole recipe, Table S10: HPNF breakfast casserole recipe, Table S11: NPHF breakfast casserole recipe, Table S12: HPHF breakfast casserole recipe, Table S13: NPNF breakfast quiche recipe, Table S14: HPNF breakfast quiche recipe, Table S15: NPHF breakfast quiche recipe, Table S16: HPHF breakfast quiche recipe: Table S17: Non-breakfast energy and macronutrient distribution, Table S18: Daily energy and macronutrient distribution.
