# Supplementary material for: Effects of Higher Dietary Protein and Fiber Intakes at Breakfast on Postprandial Glucose, Insulin, and 24-h Interstitial Glucose in Overweight Adults
Source: Nutrients. 2017 Apr 2;9(4):352. doi: 10.3390/nu9040352 (PMC5409691; doi:10.3390/nu9040352)
Supplement: Supplementary file 1 [file nutrients-09-00352-s001.pdf]

nutrients

Supplementary Material

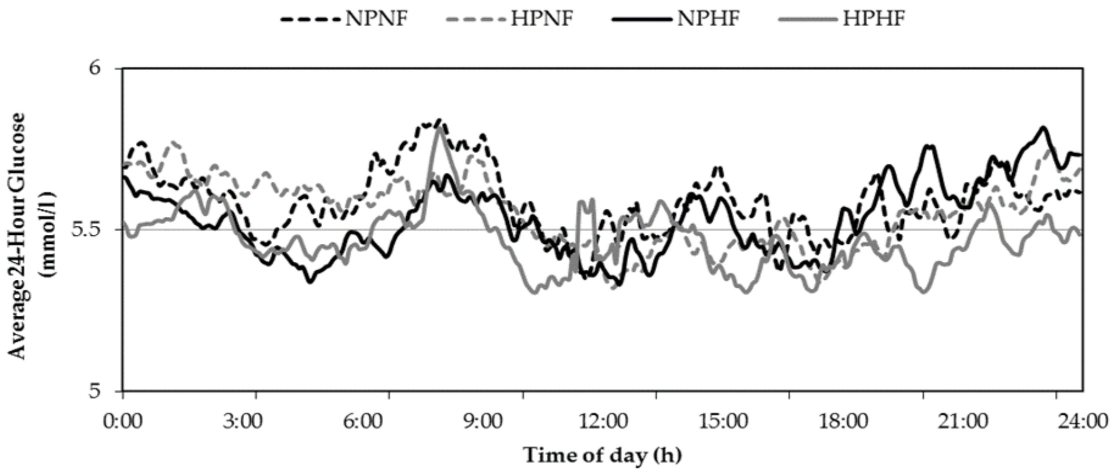

Figure S1: 24-hour time course interstitial glucose from CGM to breakfast treatments and self-selected intakes after provided breakfast. n = 19, Estimates are unadjusted means. NPNF: Normal Protein + Normal Fiber; HPNF: High Protein + Normal Fiber; NPHF: Normal Protein + High Fiber; HPHF: High Protein + High Fiber.

Table S1: NPNF breakfast burrito recipe

Food Description	Energy (kcal)	Protein (g)	Total Fat (g)	Carbohydrate (g)	Total Fiber (g)	Water (g)
Canadian Bacon Nat'l Hickory Smoked	16	2.72	0.27	0	0	
Shredded Cheddar Cheese, 2% milkfat, Mild	38.52	3	2.57	0	0	
Pure Canola Oil	51.43	0	6	0	0	
Flour Tortillas - small one	150	3.75	3.13	27.5	1.25	
Metamucil Fiber Supplement	30.6	0	0	7.34	6.12	
Egg, whole, raw	32.89	2.89	2.29	0.18	0	17.44
Apple juice	47	0.06	0.11	11.68	0.1	87.93
Seasoning Blend	18.97			3.8	0.57	
Thick & Chunky Salsa	1.25	0	0	0.31	0.16	4.43
Breakfast Total	386.66	12.42	14.36	50.81	8.2	109.81
Diet Total	386.66	12.42	14.36	50.81	8.2	109.81

Table S2: HPNF breakfast burrito recipe

Burrito<br>Food Description	Energy (kcal)	Protein (g)	Total Fat (g)	Carbohydrate (g)	Total Fiber (g)	Water (g)
Canadian Bacon Nat'l Hickory Smoked		0	0	0	0	0
Shredded Cheddar Cheese, 2% milkfat, Mild		0	0	0	0	0
Pure Canola Oil		1.8	0	0.21	0	0
Flour Tortillas - small one		120	3	2.5	22	1
Metamucil Fiber Supplement		4.74	0	0	1.14	0.95
Egg, whole, raw	57.64	108.68	9.56	7.55	0.59	0
Apple juice	176.74	94.47	0.12	0.22	23.48	0.2
Egg White Protein		25.13	5.36	0	0.3	0
Cheddar Fat Free Shredded Cheese		0	0	0	0	0
Fat Free Shredded Natural Cheddar Cheese		35.36	7.07	0	1.57	0
Seasoning Blend		6.32			1.27	0.19
Breakfast Total	234.38	396.5	25.11	10.49	50.34	2.34
Diet Total	234.38	396.5	25.11	10.49	50.34	2.34

Table S3: NPHF breakfast burrito recipe

Food Description	Energy (kcal)	Protein (g)	Total Fat (g)	Carbohydrate (g)	Total Fiber (g)	Water (g)
Canadian Bacon Nat'l Hickory Smoked	16	2.72	0.27	0	0	
Shredded Cheddar Cheese, 2% milkfat, Mild	38.52	3	2.57	0	0	
Pure Canola Oil	62.57	0	7.3	0	0	
Flour Tortillas - small one	150	3.75	3.13	27.5	1.25	
Metamucil Fiber Supplement	0.86	0	0	0.21	0.17	
Egg, whole, raw	32.89	2.89	2.29	0.18	0	17.44
Apple juice	75.2	0.1	0.18	18.69	0.16	140.69
Seasoning Blend	18.97			3.8	0.57	
Thick & Chunky Salsa	1.25	0	0	0.31	0.16	4.43
Breakast Total	396.26	12.46	15.72	50.69	2.31	162.57
Diet Total	396.26	12.46	15.72	50.69	2.31	162.57

Table S4: HPHF breakfast burrito recipe

Food Description	Energy (kcal)	Protein (g)	Total Fat (g)	Carbohydrate (g)	Total Fiber (g)	Water (g)
Canadian Bacon Nat'l Hickory Smoked	0	0	0	0	0	
Shredded Cheddar Cheese, 2% milkfat, Mild	0	0	0	0	0	
Pure Canola Oil	0	0	0	0	0	
Flour Tortillas - small one	120	3	2.5	22	1	
Metamucil Fiber Supplement	34.48	0	0	8.28	6.9	
Egg, whole, raw	108.68	9.56	7.55	0.59	0	57.64
Apple juice	56.4	0.07	0.13	14.02	0.12	105.52
Egg White Protein	25.13	5.36	0	0.3	0	
Cheddar Fat Free Shredded Cheese	0	0	0	0	0	
Fat Free Shredded Natural Cheddar Cheese	35.36	7.07	0	1.57	0	
Pictsweet Seasoning Blend	6.32			1.27	0.19	
Breakfast Total	386.37	25.06	10.19	48.02	8.21	163.15
Diet Total	386.37	25.06	10.19	48.02	8.21	163.15

Table S5: NPNF breakfast sandwich recipe

Food Description	Energy (kcal)	Protein (g)	Total Fat (g)	Carbohydrate (g)	Total Fiber (g)	Water (g)
Shredded Cheddar Cheese, 2% milkfat, Mild	38.52	3	2.57	0	0	
Pure Canola Oil	77.14	0	9	0	0	
Metamucil Fiber Supplement	0.86	0	0	0.21	0.17	
Egg, whole, raw	22.88	2.01	1.59	0.12	0	12.13
Orange Juice, 10-oz.	95.86	1.37	0	22.6	0	
Multi-Grain English Muffins	150	5	2.5	27	2	
Pork, cured, ham, extra lean (approximately 4% fat), canned, unheated	8.4	1.29	0.32	0	0	5.15
Breakfast Total	393.67	12.68	15.98	49.93	2.17	17.28
Diet Total	393.67	12.68	15.98	49.93	2.17	17.28

Table S6: NPHP breakfast sandwich recipe

Food Description	Energy (kcal)	Protein (g)	Total fat (g)	Carbohydrate (g)	Total Fiber (g)	Water (g)
Shredded Cheddar Cheese, 2% milkfat, Mild	38.52	3	2.57	0	0	
Pure Canola Oil	66	0	7.7	0	0	
Metamucil Fiber Supplement	31.9	0	0	7.66	6.38	
Egg, whole, raw	22.88	2.01	1.59	0.12	0	12.13
Orange Juice, 10-oz	66.44	0.95	0	15.66	0	
Multi-Grain English Muffins	150	5	2.5	27	2	
Pork, cured, ham, extra lean (approximately 4% fat), canned, unheated	10.8	1.66	0.41	0	0	6.62
Breakfast Total	386.53	12.63	14.77	50.44	8.38	18.75
Diet Total	386.53	12.63	14.77	50.44	8.38	18.75

Table S7: NPHF breakfast sandwich recipe

Food Description	Energy (kcal)	Protein (g)	Total Fat (g)	Carbohydrate (g)	Total Fiber (g)	Water (g)
Shredded Cheddar Cheese, 2% milkfat, Mild	51.36	4	3.42	0	0	
Pure Canola Oil	0	0	0	0	0	
Metamucil Fiber Supplement	0.86	0	0	0.21	0.17	
Egg, whole, raw	50.05	4.4	3.48	0.27	0	26.54
Orange Juice, 10-oz	92.54	1.32	0	21.81	0	
Multi-Grain English Muffins	150	5	2.5	27	2	
Pork, cured, ham, extra lean (approximately 4% fat), canned, unheated	10.8	1.66	0.41	0	0	6.62
Egg White Protein	37.5	8	0	0.45	0	
Breakfast Total	393.11	24.39	9.81	49.74	2.17	33.16
Diet Total	393.11	24.39	9.81	49.74	2.17	33.16

Table S8: HPHF breakfast sandwich recipe

Food Description	Energy (kcal)	Protein (g)	Total fat (g)	Carbohydrate (g)	Total Fiber (g)	Water (g)
Shredded Cheddar Cheese, 2% milkfat, Mild	25.68	2	1.71	0	0	
Pure Canola Oil	0	0	0	0	0	
Metamucil Fiber Supplement	31.9	0	0	7.66	6.38	
Egg, whole, raw	50.05	4.4	3.48	0.27	0	26.54
Orange Juice, 10-oz	64.07	0.92	0	15.1	0	
Multi-Grain English Muffins	150	5	2.5	27	2	
Pork, cured, ham, extra lean (approximately 4% fat), canned, unheated	27.6	4.25	1.05	0	0	16.91
Egg White Protein	37.5	8	0	0.45	0	
Breakfast Total	386.79	24.57	8.74	50.48	8.38	43.45
Diet Total	386.79	24.57	8.74	50.48	8.38	43.45

Table S9: NPNF breakfast casserole recipe

Food Description	Energy (kcal)	Protein (g)	Total Fat (g)	Carbohydrate (g)	Total Fiber (g)	Water (g)
Brunch Casserole - Egg Study	134.9	9.64	8.81	1.98	0.05	
Frozen Potatoes	38.82	0.78	1.55	8.54		
Egg, white only, raw	0	0	0	0	0	0
Spinach, cooked, from fresh, fat not added in cooking	2.3	0.29	0.03	0.37	0.24	9.07
Apple Juice 10 oz bottle	99.75	0	0	24.99	0	
Metamucil Fiber Supplement	7.33	0	0	1.76	1.47	
Pure Canola Oil	44.57	0	5.2	0	0	
White Bread	65.75	1.75	0.66	12.25	0.44	
Breakfast Total	393.42	12.47	16.25	49.89	2.19	9.07
Diet Total	393.42	12.47	16.25	49.89	2.19	9.07

Table S10: HPNF breakfast casserole recipe

Food Description	Energy (kcal)	Protein (g)	Total Fat (g)	Carbohydrate (g)	Total Fiber (g)	Water (g)
Brunch Casserole - Egg Study	118.45	8.47	7.74	1.74	0.04	
Frozen Potatoes	38.82	0.78	1.55	8.54		
Egg, white only, raw	16.8	3.82	0.06	0.26	0	30.65
Spinach, cooked, from fresh, fat not added in cooking	2.3	0.29	0.03	0.37	0.24	9.07
Apple Juice 10 oz bottle	99.75	0	0	24.99	0	
Metamucil Fiber Supplement	6.47	0	0	1.55	1.29	
Pure Canola Oil	0	0	0	0	0	
White Bread	65.75	1.75	0.66	12.25	0.44	
Egg White Protein	45	9.6	0	0.54	0	
Breakfast Total	393.34	24.7	10.03	50.24	2.01	39.72
Diet Total	393.34	24.7	10.03	50.24	2.01	39.72

Table S11: NPHF breakfast casserole recipe

Food Description	Energy (kcal)	Protei n (g)	Total Fat (g)	Carbohydrate (g)	Total Fiber (g)	Water (g)
Brunch Casserole - Egg Study	134.9	9.64	8.81	1.98	0.05	
Frozen Potatoes O'Brien	38.82	0.78	1.55	8.54		
Egg, white only, raw	0	0	0	0	0	0
Spinach, cooked, from fresh, fat not added in cooking	2.3	0.29	0.03	0.37	0.24	9.07
Apple Juice 10 oz bottle	71.25	0	0	17.85	0	
Metamucil Fiber Supplement	38.36	0	0	9.21	7.67	
Pure Canola Oil	35.14	0	4.1	0	0	
White Bread	65.75	1.75	0.66	12.25	0.44	
Breakfast Total	386.53	12.47	15.15	50.2	8.4	9.07
Diet Total	386.53	12.47	15.15	50.2	8.4	9.07

Table S12: HPHF breakfast casserole recipe

Food Description	Energy (kcal)	Protein (g)	Total Fat (g)	Carbohydrate (g)	Total Fiber (g)	Water (g)
Brunch Casserole - Egg Study	118.45	8.47	7.74	1.74	0.04	
Frozen Potatoes	44.12	0.88	1.76	9.71		
Egg, white only, raw	16.80	3.81	0.06	0.26	0	30.65
Spinach, cooked, from fresh, fat not added in cooking	2.30	0.30	0.03	0.37	0.24	9.07
Apple Juice 10 oz bottle	68.88	0	0	17.25	0	
Metamucil Fiber Supplement	38.36	0	0	9.21	7.67	
Pure Canola Oil	0	0	0	0	0	
White Bread	52.60	1.40	0.53	9.80	0.35	
Egg White Protein	45.00	9.60	0	0.54	0	
Breakfast Total	386.50	24.46	10.11	48.87	8.30	39.72
Diet Total	386.50	24.46	10.11	48.87	8.30	39.72

Table S13: NPNF breakfast quiche recipe

Food Description	Energy (kcal)	Protein (g)	Total Fat (g)	Carbohydrate (g)	Total Fiber (g)	Water (g)
Quiche Cups - 10% Protein	63.56	4.83	3.31	3.87	0.51	62.15
Cracker Crumbs	126.09	2.52	2.52	21.43	1.26	
Butter, salted	86.04	0.1	9.73	0.01	0	1.9
Orange Juice, 10-oz.	99.66	1.42	0	23.49	0	
Metamucil Fiber Supplement	2.59	0	0	0.62	0.52	
Egg, white, raw, fresh	0	0	0	0	0	0
Egg Beaters, Original	18.46	3.64	0	0.56	0	
Egg White Protein	0	0	0	0	0	
Breakfast Total	396.39	12.51	15.57	49.98	2.29	64.05
Diet Total	396.39	12.51	15.57	49.98	2.29	64.05

Table S14: HPNF breakfast quiche recipe

Food Description	Energy (kcal)	Protein (g)	Total Fat (g)	Carbohydrate (g)	Total Fiber (g)	Water (g)
Quiche Cups - 10% Protein	80.51	6.12	4.2	4.9	0.64	78.72
Cracker Crumbs	121.74	2.43	2.43	20.7	1.22	
Butter, salted	20.08	0.02	2.27	0	0	0.44
Orange Juice, 10-oz.	94.92	1.36	0	22.37	0	
Metamucil Fiber Supplement	1.29	0	0	0.31	0.26	
Egg, white, raw, fresh	0	0	0	0	0	0
Egg Beaters, Original	40.72	8.02	0	1.23	0	
Egg White Protein	37.5	8	0	0.45	0	
Breakfast Total	396.75	25.96	8.9	49.96	2.12	79.16
Diet Total	396.75	25.96	8.9	49.96	2.12	79.16

Table S15: NPHF breakfast quiche recipe

Food Description	Energy (kcal)	Protein (g)	Total Fat (g)	Carbohydrate (g)	Total Fiber (g)	Water (g)
Quiche Cups - 10% Protein	63.56	4.83	3.31	3.87	0.51	62.15
Cracker Crumbs	91.3	1.83	1.83	15.52	0.91	
Butter, salted	78.87	0.09	8.92	0.01	0	1.75
Orange Juice, 10-oz.	94.92	1.36	0	22.37	0	
Metamucil Fiber Supplement	34.48	0	0	8.28	6.9	
Egg, white, raw, fresh	0	0	0	0	0	0
Egg Beaters, Original	22.81	4.49	0	0.69	0	
Egg White Protein	0	0	0	0	0	
Breakfast Total	385.94	12.6	14.06	50.74	8.32	63.89
Diet Total	385.94	12.6	14.06	50.74	8.32	63.89

Table S16: HPHF breakfast quiche recipe

Food Description	Energy (kcal)	Protein (g)	Total Fat (g)	Carbohydrate (g)	Total Fiber (g)	Water (g)
Quiche Cups - 10% Protein	80.51	6.12	4.2	4.9	0.64	78.72
Cracker Crumbs	78.26	1.57	1.57	13.3	0.78	
Butter, salted	21.51	0.03	2.43	0	0	0.48
Orange Juice, 10-oz. To-Go Bottles	94.92	1.36	0	22.37	0	
Metamucil Fiber Supplement	33.62	0	0	8.07	6.72	
Egg, white, raw, fresh	0	0	0	0	0	0
Egg Beaters, Original	40.72	8.02	0	1.23	0	
Egg White Protein	37.5	8	0	0.45	0	
Breakfast Total	387.04	25.09	8.19	50.33	8.15	79.19
Diet Total	387.04	25.09	8.19	50.33	8.15	79.19

Table S17: Non-breakfast energy and macronutrient distribution

Dietary Variables	Breakfast Treatment			
	NPNF<br>n=17	HPNF<br>n=18	NPHF<br>n=16	HPHF<br>n=16
Energy (kcal)	1782 ± 711	1697 ± 572	1634 ± 539	1521 ± 595
Available Carbohydrate (g)	187 ±7 8	188 ± 70	171 ± 80	169 ± 73
Sugar (g)	86 ± 51	72 ± 48	82 ± 50	89 ± 48
Total Fiber (g)	16 ± 10	15 ± 5	15 ± 9	16 ± 9
Soluble Fiber (g)	7 ± 0.3	5 ± 0.3	7 ± 0.3	5 ± 0.3
Insoluble Fiber (g)	9 ± 8	10 ± 3	9 ± 5	12 ± 7
Total Protein (g)	73 ± 28	69 ± 27	65 ± 25	78 ± 27
Total Fat (g)	71 ± 37	65 ± 27	69 ± 23	57 ± 29
Saturated Fat (g)	24 ± 9	22 ± 10	22 ± 8	21 ± 11
Monounsaturated Fat (g)	26 ± 19	22 ± 10	24 ± 9	21 ± 10
Polyunsaturated Fat (g)	16 ± 0.3	17 ± 0.3	18 ± 0.3	12 ± 0.3
Trans Fat (g)	3 ± 1	2 ± 1	3 ± 1	3 ± 2
Sodium (mg)	2898 ± 1095	2777 ± 1300	2269 ± 850	2505 ± 1093

Values are means ± standard deviation (SD)

Table S18: Daily energy and macronutrient distribution

	Breakfast treatment			
Dietary variables	NPNF<br>n=17	HPNF<br>n=18	NPHF<br>n=16	HPHF<br>n=16
Energy (kcal)	2178 ± 711	2094 ± 572	2021 ± 539	1907 ± 595
Available Carbohydrate (g)	238 ± 78	238 ± 70	222 ± 80	217 ± 73
Sugar (g)	104 ± 51	94 ± 48	93 ± 50	103 ± 48
Total Fiber (g)	18 ± 10[a,b]	17 ± 5[a]	23 ± 9[a,b]	24 ± 9[b]
Soluble Fiber (g)	7 ± 0.3[a]	6 ± 0.3[a]	13 ± 0.3[b]	12 ± 0.3[b]
Insoluble Fiber (g)	11 ± 8	11 ± 3	11 ± 5	13 ± 7
Total Protein (g)	86 ± 28	94 ± 27	77 ± 25	91 ± 27
Total Fat (g)	87 ± 37	75 ± 27	83 ± 23	67 ± 29
Saturated Fat (g)	28 ± 9	25 ± 10	26 ± 8	24 ± 11
Monounsaturated Fat (g)	32 ± 19	25 ± 10	30 ± 9	24 ± 10
Polyunsaturated Fat (g)	19 ± 0.3[b]	18 ± 0.3[a,b]	20 ± 0.3[b]	13 ± 0.3[a]
Trans Fat (g)	3 ± 1	2 ± 1	3 ± 1	3 ± 2
Sodium (mg)	3665 ± 1095	3500 ± 1300	3034 ± 850	3225 ± 1093

Values are means ± standard deviation (SD). Values without a common letter are different, $p < 0.05$
